# The WAVE3-YB1 interaction regulates cancer stem cells activity in breast cancer

**DOI:** 10.18632/oncotarget.22009

**Published:** 2017-10-24

**Authors:** Kamila Bledzka, Barbara Schiemann, William P. Schiemann, Paul Fox, Edward F. Plow, Khalid Sossey-Alaoui

**Affiliations:** ^1^ Department of Molecular Cardiology, Lerner Research Institute, Cleveland Clinic, Cleveland, Ohio, USA; ^2^ Case Comprehensive Cancer Center, Cleveland, Ohio, USA; ^3^ Department of Cellular and Molecular Medicine, Cleveland, Ohio, USA

**Keywords:** WAVE3, CSCs, YB1, TNBC, chemoresistance

## Abstract

Resistance to therapy is the main cause of tumor recurrence and metastasis and cancer stem cells (CSCs) play a crucial role in this process, especially in triple-negative breast cancers (TNBCs). Unfortunately, no FDA-approved treatment is currently available for this subtype of BC, which explains the high rate of mortality in patients with TNBC tumors. WAVE3, a member of the WASP/WAVE actin-cytoskeleton remodeling family of protein, has been established as a major driver of tumor progression and metastasis of several solid tumors, including those originating in the breast. Our recently published studies found WAVE3 to mediate the process of chemoresistance in TNBCs. The molecular mechanisms whereby WAVE3 regulates chemoresistance in TNBC tumors remains largely unknown, as does the role of WAVE3 in CSC maintenance. Here we show that WAVE3 promotes CSC self-renewal and regulates transcription of CSC-specific genes, which, in part, provides a mechanistic explanation for the function of WAVE3 in chemoresistance in TNBCs. Our data show that WAVE3 is enriched in the CSC-subpopulation of TNBC cell lines. Knockout of WAVE3 via CRISPR/Cas9 significantly attenuates the CSC-subpopulation and inhibits transcription of CSC transcription factors. Mechanistically, we established a link between WAVE3 and the Y-box-binding protein-1 (YB1), a transcription factor and CSC-maintenance gene. Indeed, the interaction of WAVE3 with YB1 is required for YB1 translocation to the nucleus of cancer cells, and activation of transcription of CSC-specific genes. Our findings identify a new WAVE3/YB1 signaling axis that regulates the CSC-mediated resistance to therapy and opens a new therapeutic window for TNBCs treatment.

## INTRODUCTION

Metastatic breast cancer (BC) ranks 2^nd^ as the cause of cancer-related death in women in the United States, accounting for more than 40,000 deaths annually [1]. Breast tumors are heterogeneous and comprise at least 5 genetically distinct subtypes [2–6], and as many as 10 distinct molecular subtypes [7, 8]. Tumors of the triple-negative BC (TNBC) subtype are especially lethal due to their highly metastatic behavior [9–13]. TNBCs do not express hormone receptors (ER-α and PR) and lack amplification at the ErbB2/HER2 locus [9–13]. These specific molecular characteristics of TNBCs have impeded the development of FDA-approved targeted therapies against this BC subtype. Moreover, TNBCs are also highly proficient in becoming chemoresistant and undergoing disease recurrence through mechanisms that remain incompletely understood.

WAVE3 is a member of the WASP/WAVE family of actin-cytoskeleton remodeling proteins that regulate cell shape/morphology; they also play important roles in directing cell, motility and cancer cell invasion [14–22]. We have shown that increased WAVE3 expression/activity levels enhance invasion and metastasis of human TNBCs at least in part through upregulated metalloproteinase (MMP) expression and activity, and increased formation of invadopodia and extracellular matrix (ECM) degradation [15-20, 23]. Mechanistically, we showed that oncogenic WAVE3 activity and its stimulation of EMT and metastasis is regulated by microRNAs miR-200 and miR-31, as well as by c-Abl-mediated phosphorylation in TNBCs [19-22, 24, 25]. At the clinical level, we found aberrant WAVE3 expression to be a strong indicator of human TNBC progression, as well as an accurate predictor for TNBC tumor size, stage, and lymph node metastasis [14, 26].

Collectively, these findings implicate WAVE3 as a critical mediator of TNBC development and metastatic progression.

Recently, we observed aberrant WAVE3 expression to be associated with the development of chemoresistant phenotypes in TNBCs [23, 27], a trait that is also commonly attributed to cancer stem cells (CSCs) [28–32]. Unfortunately, the molecular mechanisms operating in coupling chemoresistance of CSCs to aberrant WAVE3 activity remain to be fully elucidated. Here we establish a novel role for WAVE3 in maintaining the CSC population and regulating CSC-specific genes. We show that WAVE3 is enriched in the CSC sub-population of TNBC cell lines, and that loss of WAVE3 expression significantly reduced the CSC niche from these cell lines. We also show that WAVE3 interacts with the transcription factor and CSC-maintenance gene YB1, and that WAVE3 is required for translocation of YB1 to the nucleus to regulate expression of the CSC-specific genes. Taken together, our findings provide new inroads in understanding the role of WAVE3 in the maintenance of the CSC population that contributes to the development of the metastatic, chemo- and radio-resistance phenotypes in TNBCs.

## RESULTS

### CRISPR/Cas9-mediated knockout of WAVE3 inhibits BC cell migration, invasion and extracellular matrix degradation

**Figure 1:**
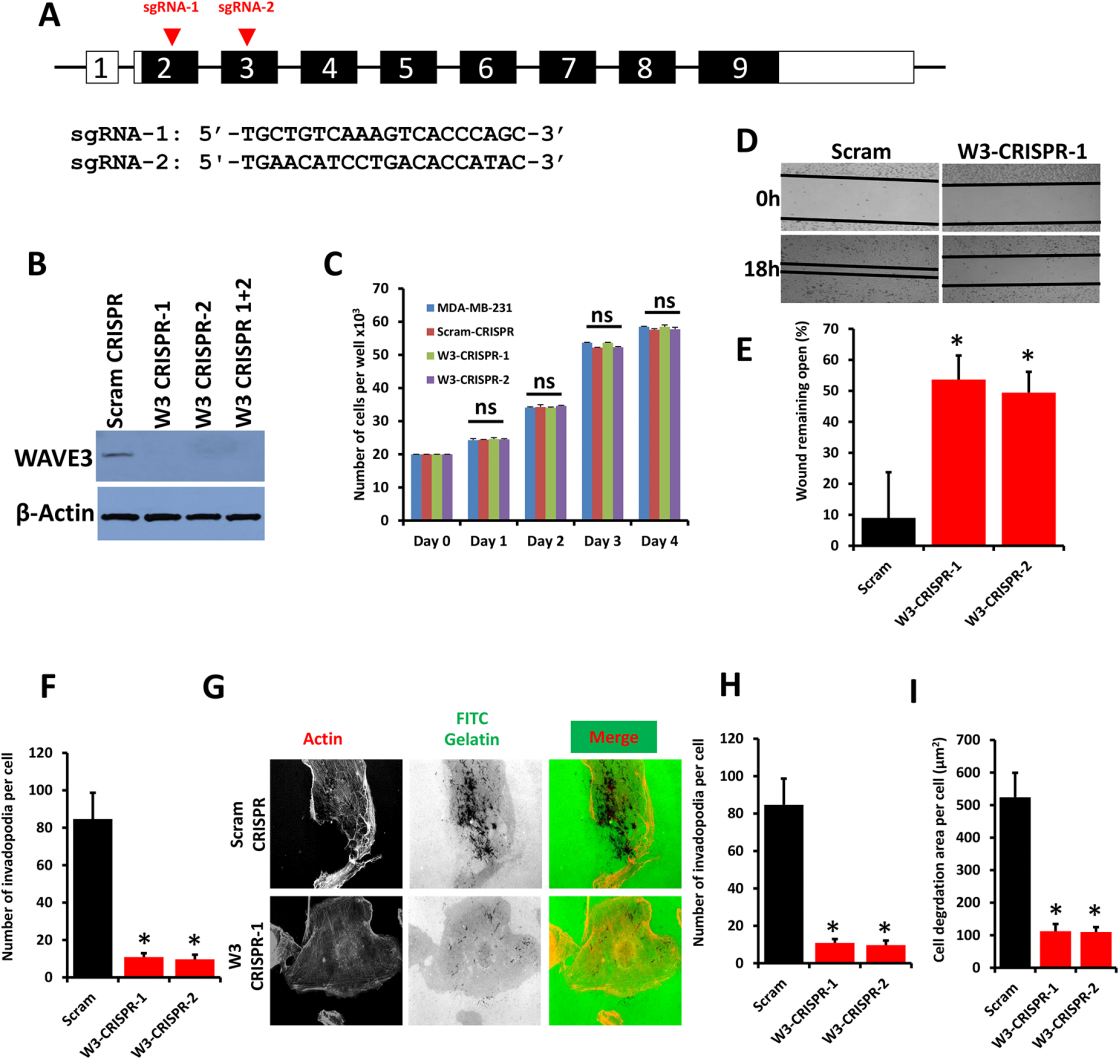
WAVE3 knockout via CRISPR/Cas9 inhibits BC cell migration and invasion *in vitro* **(A)** Genomic structure of human *WAVE3* gene showing intron-exon organization and location of sg-RNAs, (arrow-heads) in exon 2 and exon 3 of human *WAVE3* gene. **(B)** Western blots developed with anti-WAVE3 antibody of protein lysates from MDA-MB-231 transduced with a scrambled sgRNA (Scram CRISPR), sgRNA-1 (W3-CRISPR-1), sgRNA-2- (W3-CRISPR-2) or both sgRNA-1 and -2 (W3-CRISPR-1+2). β-Actin is a loading control. **(C)** Proliferation over 5 days of parental, Scram and WAVE3-deficient (W3-CRISPR-1 and -2) MDA-MB-231 cells. **(D)** Migration of Scram or WAVE3-CRISPR-1 and -2 MDA-MB-231 cells into scratch wounds in confluent monolayers over 18h. The unclosed wound (open area) at 18h from 12 different wounds was measured and plotted as the percentage of the wound at time zero **(E)**. **(F)** Invasion assays through Matrigel-coated membranes of control (Scram), W3-CRISPR-1 or -2 MDA-MB-231 cells: Invading cells were counted from six different fields and plotted as average number of invading cells per field for cells (F). **(G)** Invadopodia formation and ECM degradation assays: Control (Scram) or WAVE3-deficient (W3-CRISPR-1 and -2) MDA-MB-21 cells were seeded onto FITC-conjugated Gelatin for 18 h, at which point they were fixed and stained with phalloidin-568 to visualize actin filament. Micrographs of W3-CRISPR-1 are shown as an example (G). Invadopodia structures shown as white dots (left panels) were quantified **(H)**. Areas of ECM degradation, shown as dark spots (middle panels), coincided with invadopodia structures (right panels) and were quantified **(I)**. Data are the means ± SD, N=3; ns, not significant; ^*^, p <0.05; Student's t-test).

With the emergence of CRISPR/Cas9 technology as an efficient tool for site-specific gene editing and targeting [33, 34], we used this technology to inhibit WAVE3 expression in cancer cells. We designed two single-guide RNAs (sgRNAs), sgRNA-1 and -2, to target exon 2 and exon 3 of human WAVE3, respectively (Figure 1A) in human MDA-MB-231 BC cells, a widely used model for TNBC that has elevated levels of WAVE3 [14, 35]. These sgRNAs were very efficient in knocking out WAVE3 expression in MDA-MB-231 cells compared to cells infected with a scrambled (Scram) sgRNA (Figure 1B). Sequencing of individual PCR clones from the genomic DNA of sgRNA-targeted exon 3 of WAVE3 in MDA-MB-231 cells showed several small insertion-deletions (indels) near the target site, which resulted in in frame shifts in the coding sequence (Supplementary Figure 1).

We have previously reported on the effect of siRNA- and shRNA-mediated knockdown WAVE3 expression on cell migration and invasion in cancer cells [17, 18, 20, 21, 23, 27]. However, the effect of complete loss of WAVE3 expression using CRISPR/Cas9 has never been reported before. Therefore, having confirmed the efficiency of WAVE3 knockout using CRISPR/Cas9, we investigated the effect of WAVE3 loss on the behavior of the human MDA-MB-231 BC cells. First, we found that both the scrambled (Scram-CRISPR) and the WAVE3-sgRNAs (W3-CRISPR-1 and WAVE3-CRISPR-2, with reference to sgRNA-1 and sgRNA-2, respectively), did not have a significant effect on proliferation of MDA-MB-231 cells (Figure 1C). Next, in a wound closure assay, we found loss of WAVE3 expression (W3-CRISPR-1 and -2) in MDA-MB-231 cells resulted in a significant decrease of migration into wounds as compared to the control (Scram) cells (Figure 1D & 1E). In Boyden chamber invasion assays, less MDA-MB-231 WAVE3-deficient (W3-CRISPR-1 and -2) cells traversed the Matrigel-coated inserts compared to the Scram cells (Figure 1F). We further investigated the biological significance of loss of WAVE3 through the ability of these cancer cells to form invadopodia and degrade the extracellular matrix (ECM). MDA-MB-231 cells, like most highly invasive cancer cell lines, form invadopodia *in vitro* when seeded onto components of the extracellular matrix. Control (Scram CRISPR) or WAVE-3 deficient (W3-CRISPR-1 or -2) MDA-MB-231 cells were coated onto fluorescent gelatin-coated coverslips. After staining for F-actin, invadopodia were observed as dot-like clusters of F-actin on the ventral surface of the cells that is in direct contact with the gelatin substratum (Figure 1G, left panel). These invadopodia structures overlap with sites of degradation of the gelatin matrix (Figure 1G, middle and right panels). We found a significant reduction of both the number of invadopodia, as well as the total area of invadopodia-mediated degradation of gelatin in the WAVE3-knockout (W3-CRISPR-1 and -2) cells compared to the control (Scram CRISPR) MDA-MB-231 cells (Figure 1H & 1I, respectively). There was ∼10-fold decrease in the number of invadopodia and more than 5-fold reduction (p<0.05) in the area of ECM degradation in the W3-CRISPR cells compared to the control Scram-CRISPR cells (Figure 1H & 1I). Together, these results not only replicate our previously published findings using shRNA-mediated knockdown of WAVE3 [23, 27], but show that the effect of CRISPR/Cas9-mediated knockout of WAVE3 is more dramatic than that of shRNA-mediated knockdown, where residual WAVE3 may still has a positive effect on cell migration, invasion and invadopodia-mediated ECM degradation [23, 27]. These data also support the use of CRISPR/Cas9 gene editing as a useful and powerful tool for the investigation of the role WAVE3 in cancer.

### Loss of WAVE3 expression decreases the number of CSCs and inhibits expression of CSC genes

**Figure 2:**
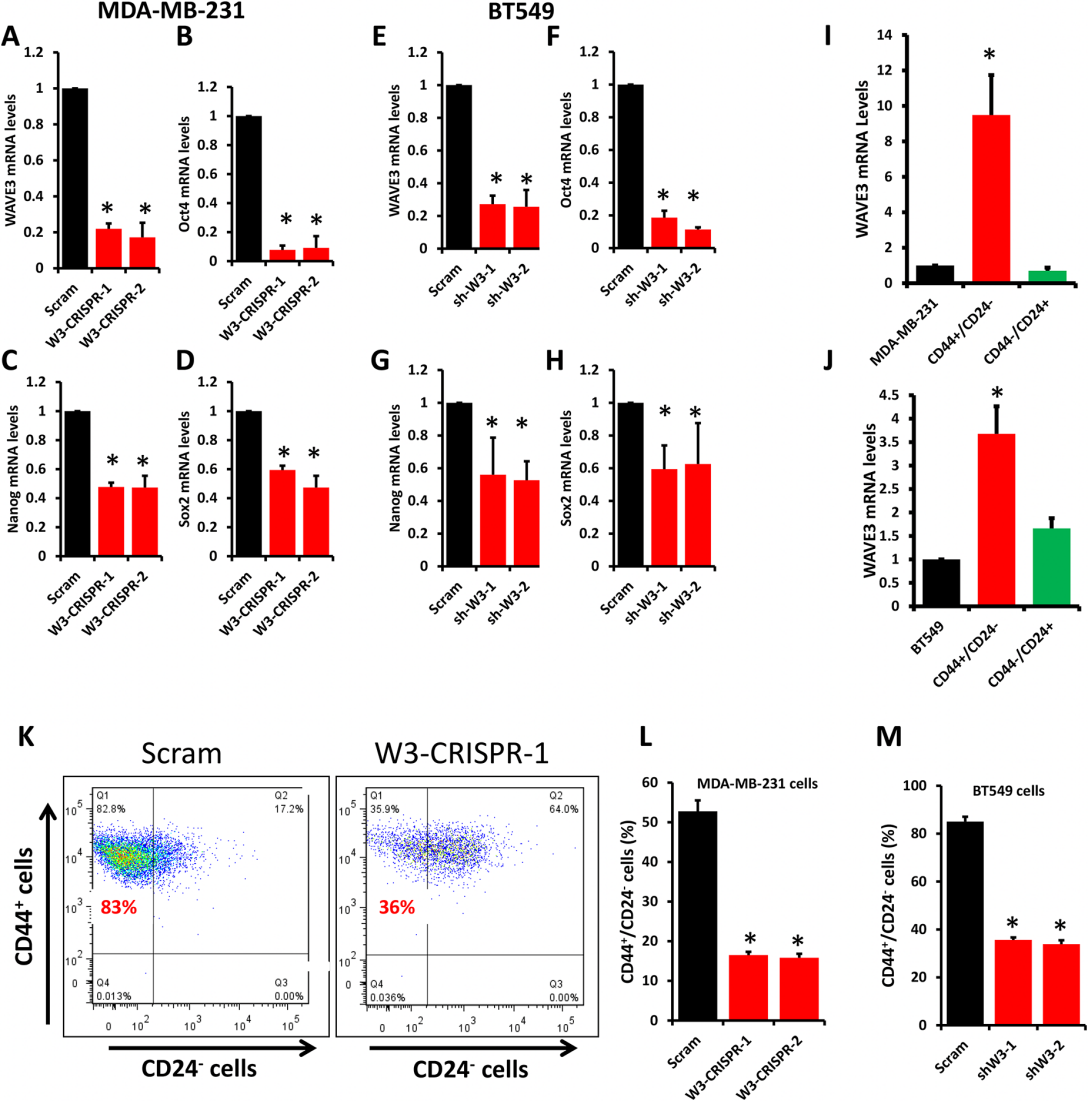
Loss of WAVE3 expression diminishes the number of cancer stem cells from the stem cell population and inhibits expression of CSC genes Quantitative-real time RT-PCR of WAVE3 **(A** & **C)**, Oct4 **(B** & **F)**, Nanog **(C** & **G)**, and Sox2 **(D** & **H)** transcripts from control (Scram) and WAVE3-deficient (W3-CRISPR-1 and -2) MDA-MB-231 cells (A to D) and BT549 cells (**E** to **H**). GAPDH was used for normalization. **(I** & **J)** Quantitative-real time RT-PCR of WAVE3 transcripts from CD44^High^/CD24^Low^ (Red bars) and CD44^Low^ /CD24^High^ (Green bars) cell subpopulations sorted from MDA-MB-231 (I) or BT549 (J) cells. Expression levels of WAVE3 were plotted as fold change to those from the parental cells (dark bars). GAPDH was used for normalization. **(K, L** & **M)** CD44^High^/CD24^Low^ cell subpopulation isolated from control (Scram) and WAVE3-deficient MDA-MB-231 (K & L) and BT549 cells. (K) Dot plot analysis of control (Scram) and WAVE3-deficient (W3-CRISPR-1) MDA-MB-231 cells. The upper left quadrant in both panels contains the CD44^High^/CD24^Low^ enriched cell population. (L & M) Quantification using flow cytometry and cell sorting analyses of the CD44^High^/CD24^Low^ cell subpopulation in control (Scram) or WAVE3-deficient (W3-CRISPR-1 and -2) MDA-MB-231 cells (L) and WAVE3-knockdown (shW3-1 and -2) BT549 (M) cells. Data are the means ± SD, N=3; ^*^, p <0.05; Student's t-test).

Tumors of the TNBC subtype have been reported to contain a subpopulation of cancer stem cells that endows them with their aggressiveness, frequent recurrence and resistance to standard of care therapeutic regimens [36, 37]. Our previously published study showed that WAVE3 expression is upregulated in several TNBC cell lines, including those of the mesenchymal stem-like (MDA-MB-231) and mesenchymal (BT549) subtypes. Our recent publications showed that rendering these TNBC cells deficient in WAVE3 expression sensitizes them to the anticancer and apoptotic activities of standard-of-care chemotherapies [23, 27]. Therefore, we investigated the effect of loss WAVE3 in these TNBC cells on their CSC populations. First, we showed that CRISPR/Ca9-mediated knockout of WAVE3 in MDA-MB-231 cells (W3-CRISPR-1 and -2, Figure 2A) and WAVE3-shRNA-knockdown in BT549 cells (sh-W3-1 and -2, Figure 2E) significantly inhibited the expression levels of Oct4 (90% loss for 231, Figure 2B; and 80% loss for BT549, Figure 2F), Nanog (60% loss for 231, Figure 2C; and 50% loss for BT549, Figure 2G), and Sox2 (∼50% loss in 231, Figure 2D; and 40% loss in BT549, Figure 2H), well-established markers of cancer stem cells. These data suggest that WAVE3 may be required for the maintenance of the CSC population in MDA-MB-231. To further test this interpretation, we used cell sorting to isolate the “stem” (*i.e.,* CD44^High^/CD24^Low^) and “non-stem” (*i.e.,* CD44^Low^/CD24^High^) populations of MDA-MB-231 and BT549 [37]. We found WAVE3 expression levels to be more than 10-fold higher in the MDA-MB-231-derived stem subpopulation compared to its non-stem counterpart (Figure 2I). These results were duplicated in the BT549 cells where WAVE3 transcript levels were ∼4-fold higher in the CD44^High^/CD24^Low^ subpopulation compared to their CD44^Low^/CD24^High^ counterparts (Figure 2J). These data correlated with the findings that the level of enrichment of the CD44^High^/CD24^Low^ subpopulation was ∼3-fold higher in the control (Scram) MDA-MB-231 than in their WAVE3-deficient (W3-CRISPR-1 and -2) counterparts (Figure 2K & 2L). Comparable results were found in BT549 cells where the CD44^High^/CD24^Low^ population was enriched more than 2-fold in the control (Scram) BT549 than in their WAVE3-deficient (W3-shRNA-1 and -2) counterparts (Figure 2M). Therefore, these data confirm the involvement of WAVE3 in the maintenance of the CSC population in these two TNBC cells.

### Loss of WAVE3 inhibits key biological functions of CSCs

**Figure 3:**
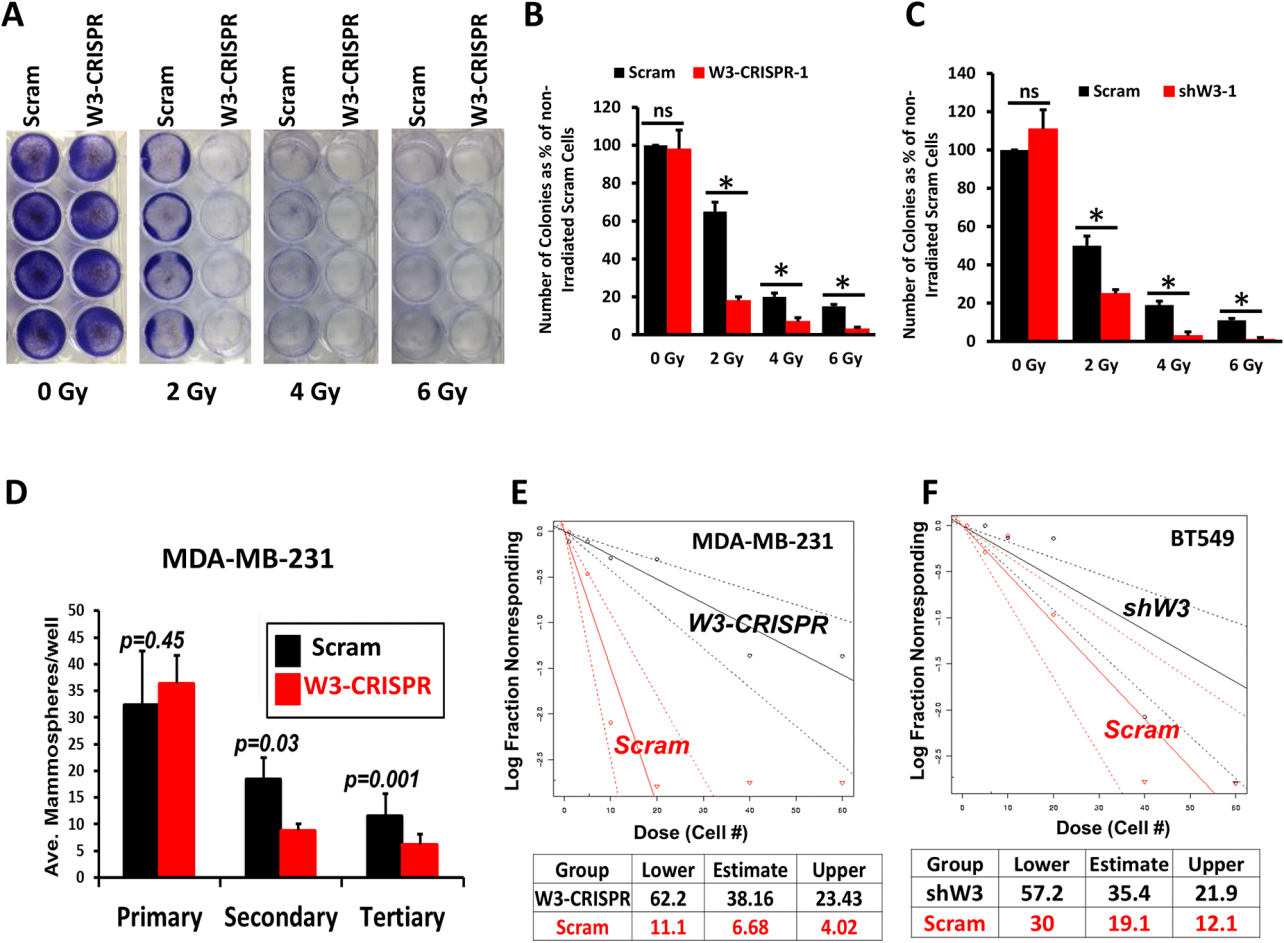
Loss of WAVE3 inhibits key biological functions of cancer stem cells **(A)** Representative micrographs of control (Scram) or WAVE3-deficient (W3-CRISPR-1) MDA-MB-231 cell colonies grown over a 2-week period in response to variable levels of gamma-ray irradiation. **(B** & **C)** Quantification of the number of colonies under the conditions shown in (A) for MDA-MB-231 cells (B) and BT549 cells (C) for. Data are the means ± SD, N=3; ^*^, p <0.05; Student's t-test). **(D)** Mammosphere formation assay. Equal number of control (Scram) and WAVE3-deficient (W3-CRISPR-1) MDA-MB-231 cells were cultured in very low adhesion 96-well culture plates over a 2-week period, and the number of colonies formed were counted and plotted as the average number of mammospheres per well from the primary, secondary and tertiary passage. **(E** & **F)** Extreme limiting dilution assay (ELDA) of control and WAVE3-deficient (W3-CRISPR-1) MDA-MB-231 cells (E) and WAVE3-knockdown (shW3-1) BT549 cells (F). Calculation of the frequency of colony-forming cells is shown under the plot.

Chemoresistance is a characteristic of CSCs [31, 38, 39]. Recent studies from our laboratory showed that inhibition of WAVE3 expression sensitized MDA-MB-231 and BT549 cells to chemotherapy [27]. Building on these finding we sought to investigate whether WAVE3 is linked to radiation resistance, also attributable to CSCs [40]. Control (Scram) or the WAVE3-knockout (W3-CRISPR-1) MDA-MB-231, or WAVE3-knocdown (shW3-1) BT549 cells were exposed to increasing radiation levels and the number of colonies formed after 2 weeks were counted. No significant difference in the number of colonies formed from the untreated control (Scram) and W3-CRISPR MDA-MB-231cells was noted (Figure 3A & 3B). However, the W3-CRISPR cells that received 2 Gy of radiation formed ∼4-fold less colonies compared to the control (Scram) cells (Figure 3A & 3B). This difference remained significant at 4 Gy and 6 Gy treatments (Figure 3A & 3B), supporting the involvement of WAVE3 in the resistance to radiation therapy. These results were replicated with the BT549 cells lacking WAVE3 expression (Figure 3C). In a mammosphere formation assay, we found that depletion of WAVE3 in MDA-MB-231 cells resulted in ∼3-fold decrease in the secondary passage (p=0.03) and ∼2-fold decrease in the tertiary passage (p=0.001) in the number of mammospheres formed by the W3-CRISPR compared to control Scram cells (Figure 3D). This finding was confirmed using the extreme limiting dilution analysis (ELDA): the frequency of MDA-MB-231 cells that were able to form mammospheres dropped from ∼1 in 10 in the control (Scram) cells to ∼1 in 60 in WAVE3-deficient cells (Figure 3E). Similar results were found with the BT549 cells where the ELDA assay indicated that the frequency dropped by 50%; 1 in 30 in Scram vs 1 in 60 in the WAVE3-sh cells (Figure 3F).

### WAVE3 modulates CSC maintenance through its interaction with Y-box binding protein-1

**Figure 4:**
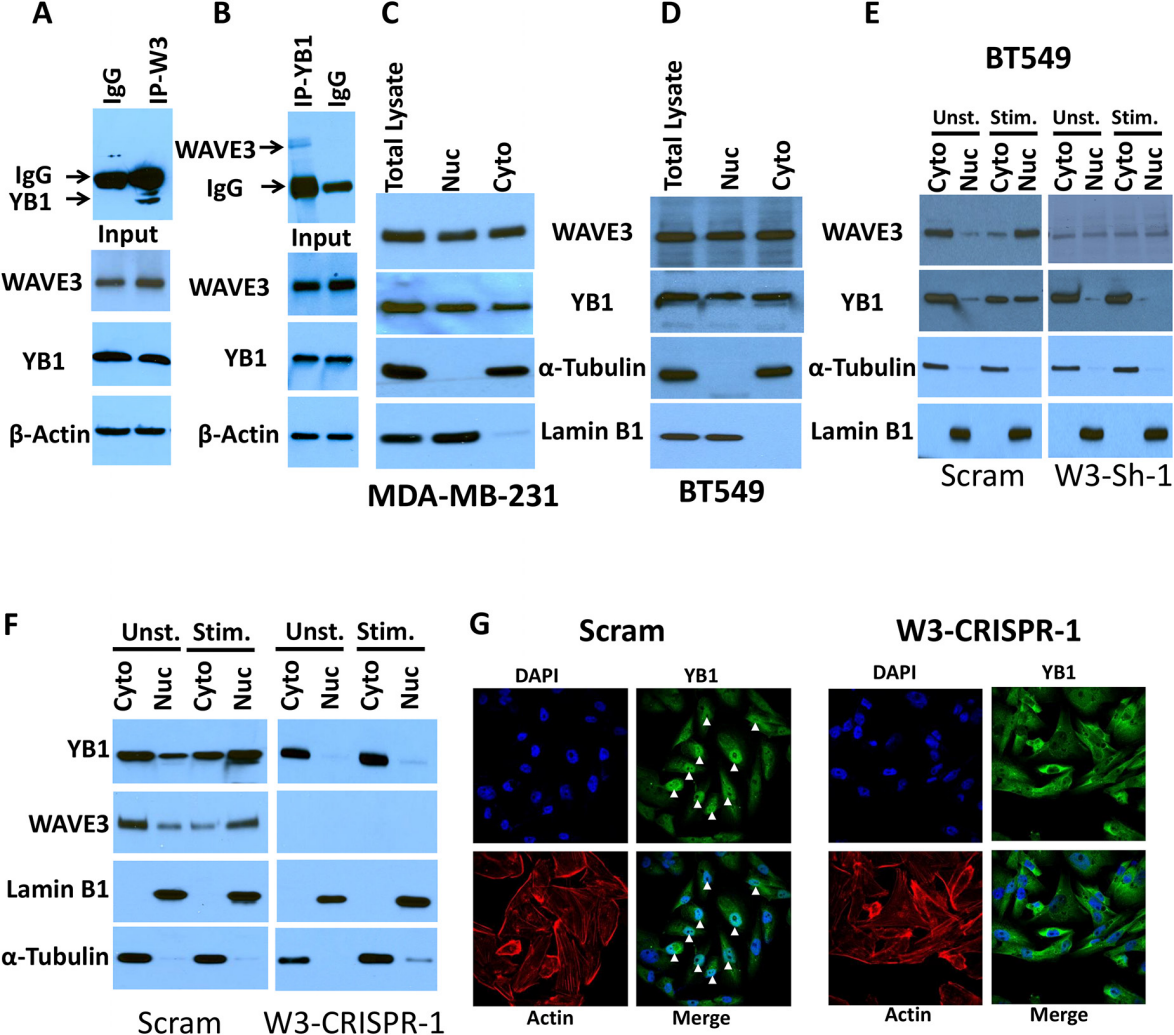
WAVE3 modulates CSC maintenance through its interaction with YB1 protein **(A** & **B)** Protein lysates prepared from MDA-MB-231 cells was used for immunoprecipitation with anti-YB1 antibody or control IgG (A); and anti-WAVE3 antibody or control IgG (B) and subjected to immunoblotting analysis with anti-WAVE3 antibody (A, upper panel) or anti YB1 antibody (B, upper panel). The results show that both WAVE3 and YB1 are present in the same protein immunocomplexes (A & B, upper panels). In control blots, cell lysates were also immunoblotted with anti-WAVE3 and anti-YB1 antibodies to show the presence of equal amounts of these proteins in the cell lysates (input panels). β-Actin is a loading control. **(C** & **D)** Immunoblot analysis of total protein lysates, the nuclear or the cytoplasmic fractions of MDA-MB-231 cells (C) and BT549 cells (D) with anti-WAVE3 and anti-YB1 antibodies. **(E** & **F)** Immunoblot analysis of total protein lysates, the nuclear or the cytoplasmic fractions of unstimulated or FBS-stimulated control (Scram) or WAVE3-knockdown (W3-Sh-1) BT549; and control (Scram) or WAVE3-deficient (W3-CRISPR-1) MDA-MB-231 cells with anti-WAVE3 and anti-YB1 antibodies. For both panels E and F, α-Tubulin and Lamin B1 were used as controls for the cytoplasmic and nuclear fractions, respectively. **(G)** Representative confocal microscopy images of control (Scram) and WAVE3-deficient (W3-CRISPR-1) MDA-MB-231 cells stained for YB1- (green) and actin filaments (Red). Nuclei were counterstained with DAPI (blue). Arrowheads point to the localization of YB1 to the nucleus.

WAVE3 was originally identified as actin-cytoskeleton remodeling protein by binding and activating the Actin-related protein complex 2 and 3 (Arp2/3) [41]. However, we and others have found WAVE3 and other members of the WAVE/WASP family of proteins to act as a scaffolding protein by binding to other protein complexes beside the Arp2/3 complex [19, 42, 43]. Accordingly, we postulated that protein-protein interactions, involving WAVE3 may be involved in maintaining CSC self-renewal. To identify such WAVE3-intercting proteins, we performed mass spectrometry analyses of isolated WAVE3 immunocomplexes from MDA-MB-231 lysates. Among several proteins identified by this approach (Supplementary Table 2) was the Y-box binding protein-1 (YB1). YB1 has been established as a CSC-specific transcription factor [44–46]. Recent studies also found YB1 to be a key factor in maintaining the CSC niche in breast and other cancers [47–49]. To verify the mass spectrometry finding (Supplementary Figure 2), we performed immunoprecipitation analysis and confirmed the presence of YB1 in the WAVE3-immunoprecipitates of MDA-MB-231 cell lysates, but not in the immunoprecipitates of the IgG control (Figure 4A). We also performed the converse immunoprecipitation analysis and found that WAVE3 was associated with YB1-immunoprecipitates from MDA-MB-231 cell lysates (Figure 4B).

YB1 can be found in the cytoplasm where it regulates mRNA translation and in the nucleus where it acts as a transcription factor that regulates expression of CSC genes [50–53]. Since we showed that WAVE3 is also involved in the maintenance of the CSC population and regulates expression of CSC-specific genes (Figure 2 & Figure 3), we sought to investigate whether WAVE3 influences the function of YB1 as a CSC-specific transcription factor in the nucleus. First, we employed several algorithms that predict the presence nuclear localization signals (NLSs) in proteins, such the cNLS Mapper, and identified two potential NLSs in the N-terminus of WAVE3 (Supplementary Figure 3). Next, we analyzed the nuclear and cytosolic fractions of MDA-MB-231 and BT549 lysates and confirmed the presence of WAVE3 in the nucleus (Figure 4C for MDA-MB-231; and Figure 4D for BT549). We also showed that, while YB1 can be found in the cytosolic and nuclear fractions of both resting and FBS-stimulated control (Scram) BT549 (Figure 4E, left panels) and MDA-MB-231 cells (Figure 4F, left panels), YB1 remains restricted to the cytosolic fraction in WAVE3-deficient BT549 (W3-Sh-1) cells (Figure 4E, right panels and Supplementary Figure 4), and WAVE3-deficient MDA-MB-231 (W3-CRISPR) cells (Figure 4F, right panels). Thus, WAVE3 is required for the YB1 translocation to the nucleus. We further confirmed the role WAVE3-YB1 interaction for the translocation of YB1 to the nucleus by immunostaining. While abundant YB1 immunostaining (green) was detected in the nuclei of control (Scram) MDA-MB-231 cells (Figure 4G), the WAVE3-KO (W3-CRISPR-1) cells show little to no YB1 signal in their nuclei (Figure 4G). Together, these data establish the important role WAVE3-YB1 interaction plays in the regulation of CSC population in BC.

### WAVE3 regulates the YB1 nuclear translocation through its proline-rich domain (PRD)

**Figure 5:**
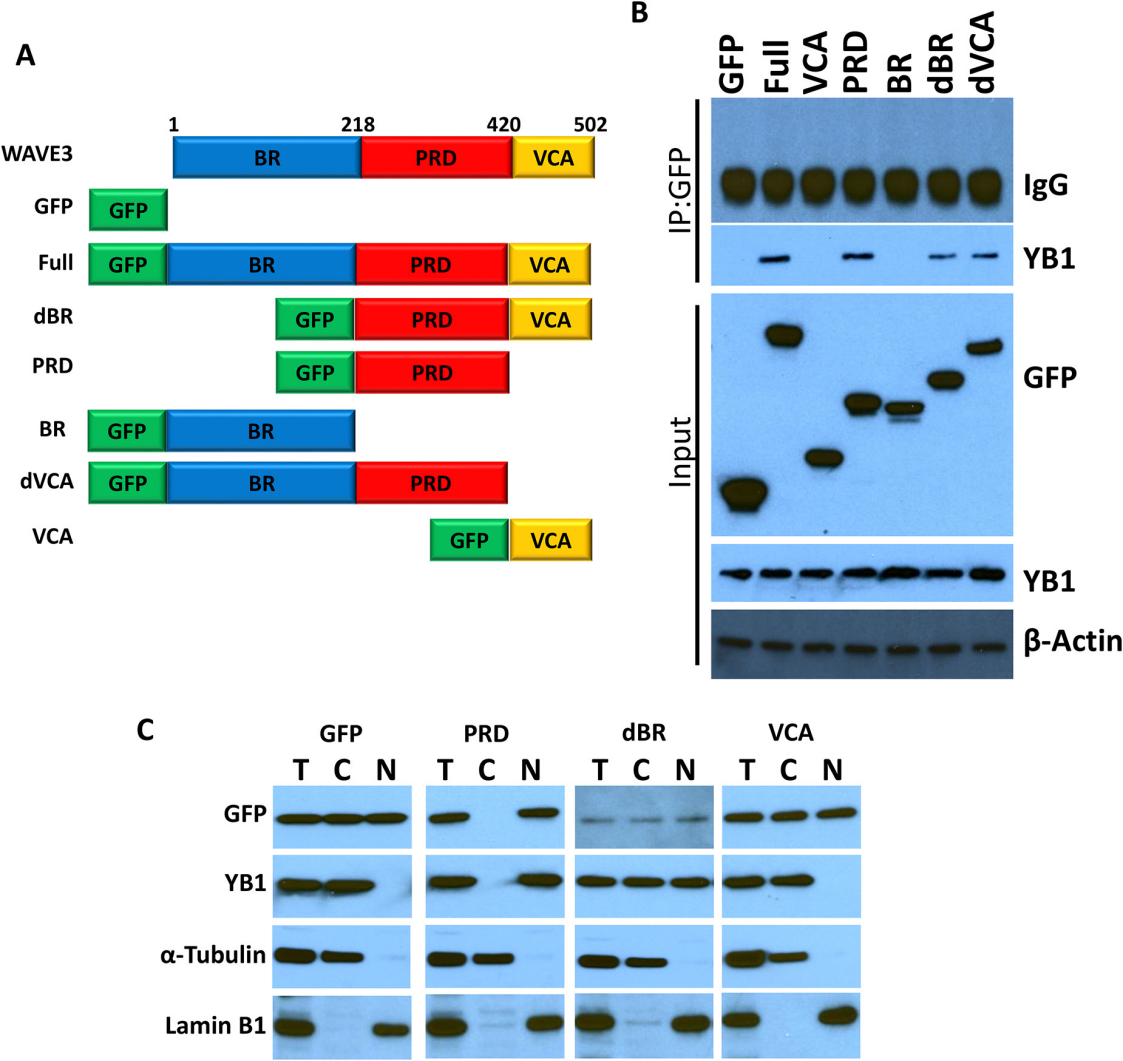
YB1 binds to the PRD domain of WAVE3 **(A)** Graphic representation of the WAVE3 functional domains and GFP-fused truncation mutants. BR: Basic Region; PRD, Proline Rich Domain; VCA, Verprolin, Coffilin and Acidic. **(B)** Proteins lysate prepared from WAVE3-deficient (W3-CRISPR-1) MDA-MB-231 cells that were transfected with the indicated constructs, were used for immunoprecipitation with anti-GFP antibody and subjected to immunoblotting analysis with anti-YB1 antibody. Cell lysates were also immunoblotted with anti-GFP and anti-YB1 antibodies to show expression of different domains of WAVE3 fused to the GFP protein in MDA-MB-231 cells and the presence of equal amounts of these proteins in the cell lysates (input panels). In all cases the size of proteins identified on the western blot are as expected for the protein fragment. β-Actin is a loading control. **(C)** Immunoblot analysis of total protein lysates (T), the nuclear (N) or the cytoplasmic (C) fractions with anti-GFP and anti-YB1 antibodies of protein lysates of FBS-stimulated WAVE3-deficient (W3-CRISPR-1) MDA-MB-231 cells that were transfected with GFP alone or GFP-fusions of WAVE3-truncation mutants. α-Tubulin and Lamin B1 were used as controls for the cytoplasmic and nuclear fractions, respectively.

**Figure 6:**
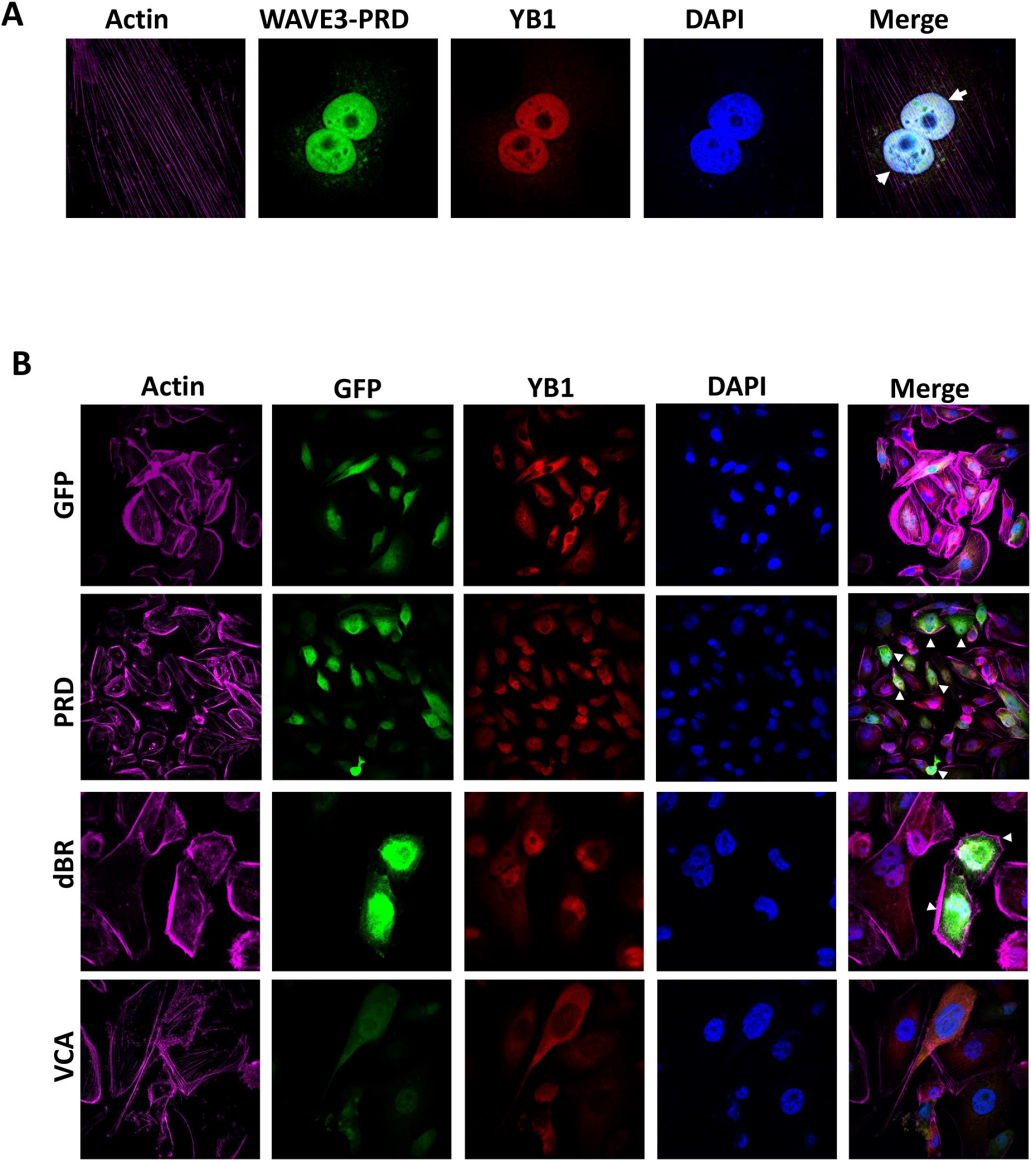
WAVE3 regulates YB1 nuclear translocation through its proline-rich domain (PRD) **(A)** Representative confocal microscopy images of WAVE3-deficient MDA-MB-231 cells that were transfected with WAVE3 PDR domain fused to the C-terminus end of GFP, were stained for YB1 (red) and actin filaments (magenta). Nuclei were counterstained with DAPI (blue). The arrowheads point to an almost-complete nuclear translocation of YB1 and WAVE3-PRD domain to the nucleus. **(B)** Representative confocal microscopy images of WAVE3-deficient MDA-MB-231 cells that were transfected with GFP alone or GFP-fusion of WAVE3-deletion mutants (green) were stained for YB1 (red) and actin filaments (magenta). Nuclei were counterstained with DAPI (blue). Arrowheads point to the nuclear co-localization of YB1 with WAVE3-PRD and WAVE3-dBR domains, but not with GFP alone or WAVE3-VCA domains.

WAVE3 is composed of several distinct subdomains (Figure 5A): *(i)* a N-terminal basic region (BR); (*ii*) a central proline-rich domain (PRD), which binds SH3-containing proteins; and *(iii)* a C-terminal verprolin-like, cofilin-like, and acidic domain (VCA) that binds and activates the Arp2/3 protein complex to initiate actin polymerization [15, 16, 41, 54–57]. We sought to establish which of these WAVE3 subdomains harbor YB1 binding site. GFP-tagged subdomains shown in Figure 5A were expressed in WAVE3-deficient (W3-CRISPR-1) MDA-MB-231 cells (Figure 5B). Cell lysates were immunoprecipitated with anti-GFP followed by immunoblotting for the YB1 protein. YB1 was in immunoprecipitates of lysates expressing full-length WAVE3 (Full; positive control), while YB1 could not be detected the immunoprecipitates of cell expressing GFP alone (negative control). YB1 protein was present in the lysates of cells expressing the PRD, WAVE3 lacking the basic region (dBR) and WAVE3 lacking the VCA (dVCA) domain, but was absent in the immunoprecipitates of lysates expressing the VCA and basic region (BR). These data suggest that the minimal region of WAVE3 responsible for YB1 binding resides in the PRD subdomain (Figure 5B). Further evidence that the PRD of WAVE3 was necessary and sufficient for the translocation of YB1 to the nucleus was sought. Western blotting of the nuclear and cytosolic fractions of MDA-MB-231 lysates expressing GFP-tagged WAVE3 and its truncation variants showed that YB1 can be found in both the nuclear and cytoplasmic fractions of lysates expressing the dBR domain, which contains the PRD. In contrast, YB1 was predominantly present in the nuclear fraction of lysates of cells the PRD alone (Figure 5C). Conversely, YB1 was confined to the cytoplasmic fraction of lysates expressing GFP alone or the VCA domain, which do not contain the PRD (Figure 5C). These findings are in full accord with immunofluorescence analyses (Figure 6), where YB1 (Red) and the PRD of WAVE3 (Green) were co-localized in the nucleus (Blue) of MDA-MB-231 cells expressing GFP-tagged PRD (Figure 6A & 6B, second panels from the top). The dBR domain also colocalized with YB1 in the nucleus, although some staining could also be detected in the cytoplasm (Figure 6B, third panels from the top). Finally, neither GFP alone (Figure 6B, top panels) nor the VCA domain (Figure 6B, bottom panels) colocalized with YB1 in the nucleus. Thus the PRD of WAVE3 is required for the nuclear translocation of YB1.

**Figure 7:**
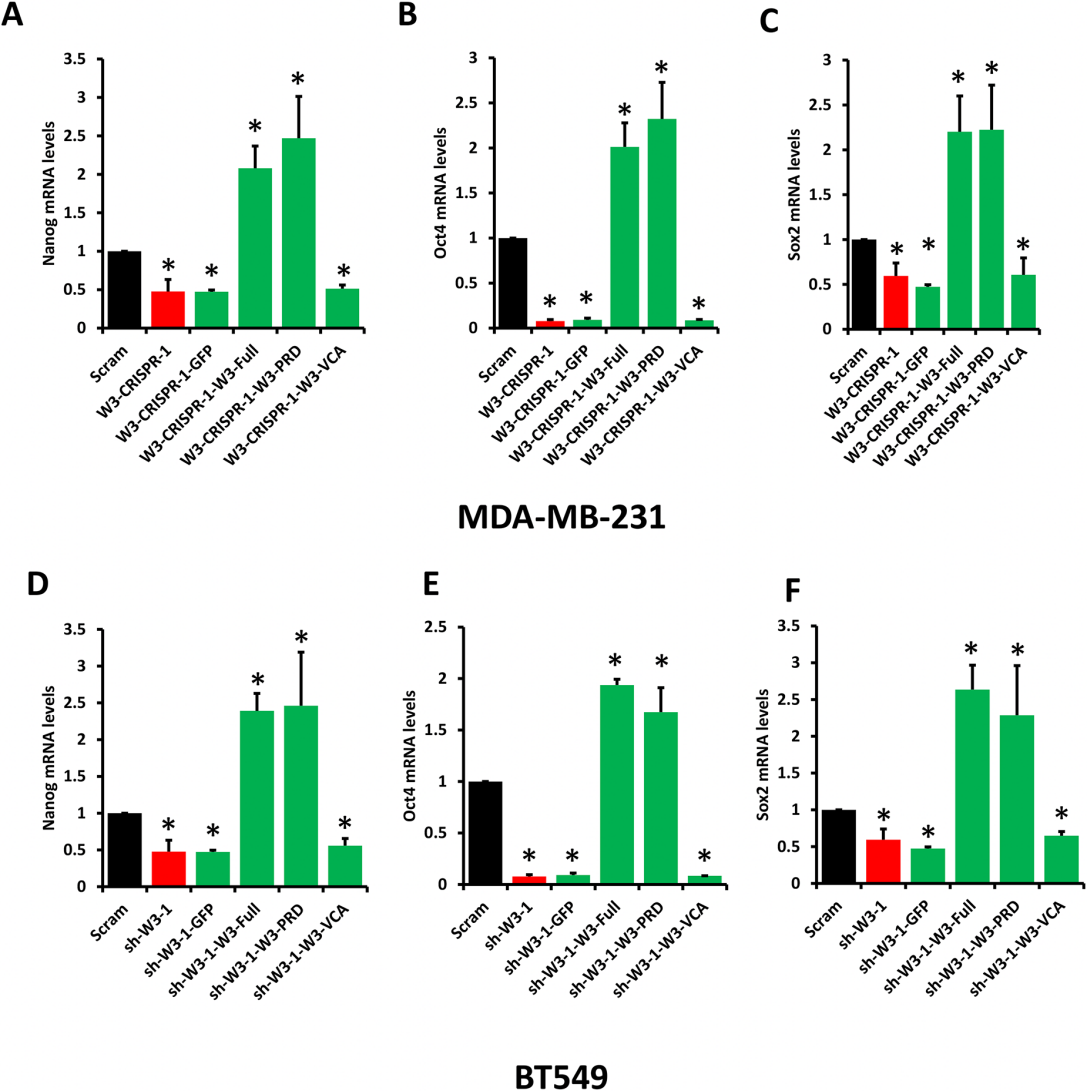
Rescue experiments WAVE3 regulates the YB1-mediated activation of transcription of CSC genes through its proline-rich domain (PRD).Quantitative-real time RT-PCR of Nanog **(A** & **D)**, Oct4 **(B** & **E)**, and Sox2 **(C** & **F)** transcripts from control (Scram) and WAVE3-deficient (W3-CRISPR-1) MDA-MB-231 cells (A to C) and WAVE3-deficient (Sh-W3-1) BT549 cells (D to F) that were transfected with the indicated expression constructs. GAPDH was used for normalization. Data are the means ± SD, N=3, ^*^, p <0.05 compared to Scram; Student’s t-test.

To further support the finding that WAVE3-YB1 interaction and the WAVE3-mediated translocation of YB1 to the nucleus are required for the maintenance of CSCs and the regulation of expression of CSC-specific genes, and are specific to WAVE3 function, we performed rescue experiments. WAVE3-deficient MDA-MB-231 (W3-CRISPR-1) and BT549 (sh-W3-1) cells were transfected with GFP-tagged subdomains of WAVE3. The transfection efficiency in MDA-MB-231 cells is shown in Figure 5B, while that of BT549 cells is shown in Supplementary Figure 5. As expected, loss of WAVE3 in either MDA-MB-231 (W3-CRISPR-1) and BT549 (sh-W3-1) cells resulted in a significant inhibition of the CSC genes Nanog (Figure 7A & 7D), Oct4 (Figure 7B & 7E), and Sox2 (Figure 7C & 7F). Overexpression of the GFP protein alone, also as expected, had no effect of the expression levels of these genes, which remained significantly lower than the in the control (Scram) cells. On the other hand, overexpression of the full-length WAVE3 (sh-W3-1-W3-Full), not only restored the expression of the CSC genes, but resulted in more than 2-fold increase in the expression levels of these genes, compared to the control (Scram) cells. Overexpression of the WAVE3-PRD domain, which we showed to bind to YB-1 (Figure 5B) and to be sufficient for the translocation of YB1 to the nucleus (Figure 5C & Figure 6), also stimulated the expression of the CSC genes by two folds or more in both MDA-MB-231 (Figure 7A–7C) and BT549 (Figure 7D–7F). Conversely, overexpression of the WAVE3 VCA domain that is not involved in the interaction with YB1 and its translocation to the nucleus, was unable to restore the expression of the CSC genes in both cell types. Thus, these rescue experiments confirm the important role of WAVE3 in the YB1-mediated maintenance of CSCs and the regulation of the CSC-specific genes.

### YB1 expression is upregulated in aggressive BC and its downregulation inhibits expression of CSC genes

**Figure 8:**
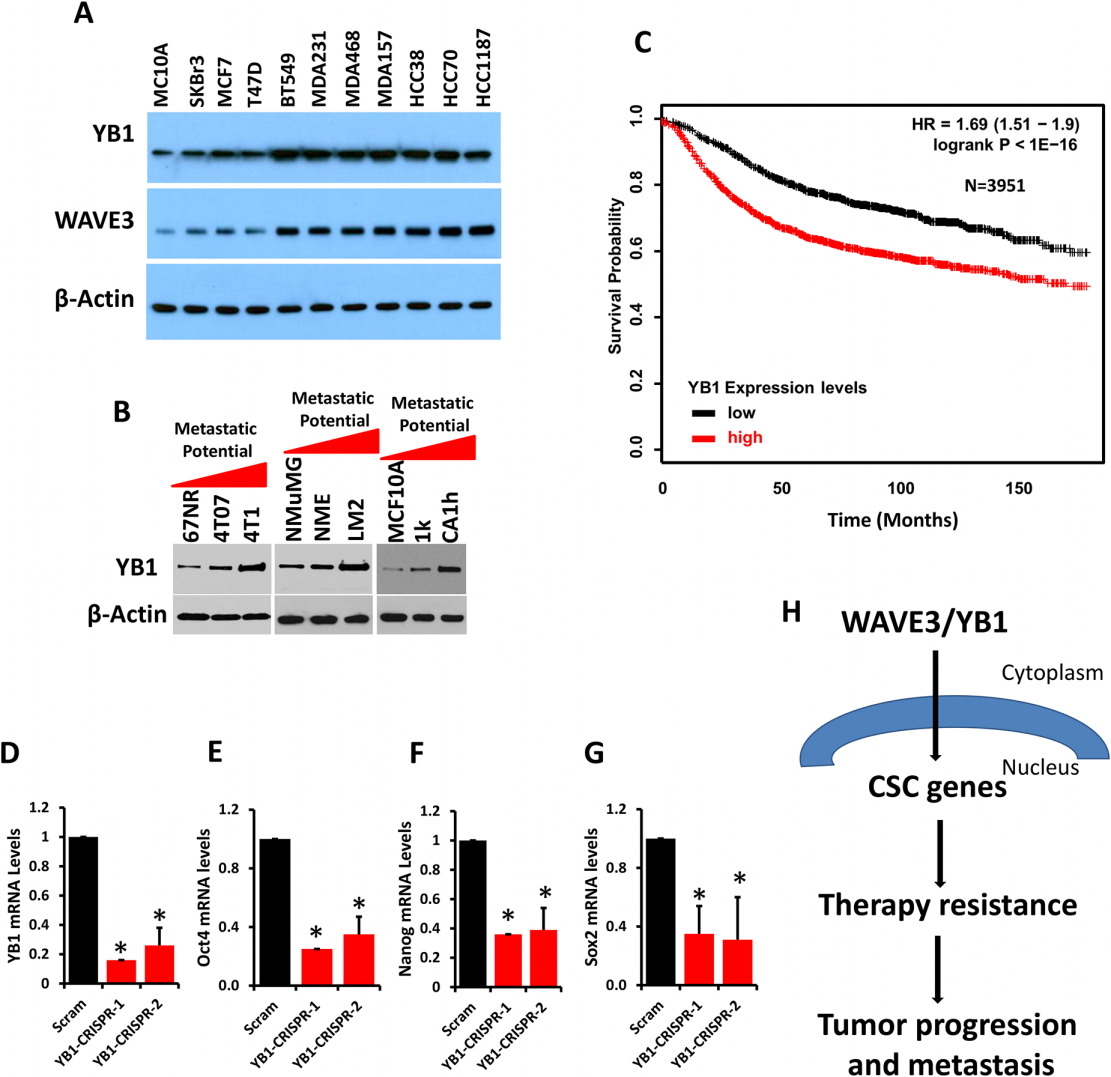
YB1 expression is upregulated in aggressive BC and its downregulation inhibits expression of CSC genes **(A)** Western blots with anti-YB1 and anti-WAVE3 antibodies on lysates of human breast cancer cell lines (A), mouse 4T1 (**B**, left panel), mouse NMuMG (B, middle panel) BC progression series, human MCF10A BC progression series (B, right panel). β-Actin is a loading control. **(C)** Kaplan-Meier (KM, http://kmplot.com/analysis/) plot correlating survival of 3951 breast cancer patients with YB1 expression levels. Quantitative-real time RT-PCR of YB1 **(D)**, Oct4 **(E)**, Nanog **(F)**, and Sox2 **(G)** transcripts from control (Scram) and YB1-deficient (YB1-CRISPR-1 and -2) MDA-MB-231 cells. GAPDH was used for normalization. Data are the means ± SD, N=3; ^*^, p <0.05; Student's t-test. **(H)** Model describing the WAVE3/YB1 interaction in the maintenance of CSCs and the regulation of expression of CSC-specific genes, which are linked to resistance to therapy and, therefore, tumor progression and metastasis.

Enhanced expression of YB1 has been reported in several human cancers [48, 49, 52, 58–62]. In order to address its role in BC, we analyzed YB1 expression across a panel of human BC cell lines. YB1 levels were several-fold higher in aggressive human BC cells; i.e. MDA-MB-231 and BT549, compared to less aggressive BC lines, T47D or MCF7 (Figure 8A, top panel). This trend follows that of WAVE3, where its expression levels correlated with the aggressive phenotype of the BC cell lines (Figure 7A, middle panel). We also found YB1 expression to be increased in metastatic murine 4T1 and non-invasive 4T07 cells relative to their indolent 67NR counterparts (Figure 8B). A similar trend was observed in the murine NMUMG BC series: YB1 expression was significantly elevated in the LM2 metastatic cells compared to their less aggressive NME cells or non-tumorigenic NMuMG counterparts (Figure 8B). Finally, YB1 was expressed at very low levels in normal human MCF10A and indolent MCF10aT1K cells relative to their high-grade and more aggressive human MCF10aCa1h counterpart (Figure 8B). Next, we addressed whether this association of YB1 expression with tumor aggressiveness also occurred in human BC tumors. We interrogated the KM-Plotter BC dataset (http://kmplot.com/analysis/) and found that increased expression levels of YB1 correlated with poor outcome and reduced survival in BC patients (Figure 8C). These findings suggest that increased YB1 expression is characteristic of invasive BC. Depletion of YB1 from the MDA-MB-231 cells via CRIPR/Cas9, using two different sgRNAs (Figure 8D), resulted in a significant decrease in the expression levels of the CSC genes Oct4 (Figure 8E), Nanog (Figure 8F), and Sox2 (Figure 8G), thereby supporting the established role of YB1 in CSC properties.

Collectively, our data confirm the involvement of both WAVE3 and YB1 in the maintenance of the CSC population and the regulation of CSC gene expression. Our observations further establish a novel role of WAVE3 in the pathological functions of CSCs in chemoresistance and, therefore tumor progression and metastasis (Figure 8H).

## DISCUSSION

CSCs are closely associated with tumor resistance to therapy, a trait that is responsible for tumor recurrence and metastasis [39]. Therefore, CSCs have been the focal point of targeting for effective cancer therapy. This study demonstrates that WAVE3 regulates CSC maintenance and does so at least in part by controlling the translocation to the nucleus of the CSC-specific transcription factor YB1. To our knowledge, this is the first study to show that WAVE3 influences CSC function, thereby providing a mechanism by which increased levels of WAVE3 in TNBCs correlates with resistance to therapy and poor prognosis [14, 23, 27], properties tightly linked to CSCs. Additionally, we show that WAVE3 is enriched in the CSC subpopulation of TNBC cells, which contributes to the activation of their invasive properties and their increased resistance to radiotherapy further promoting the neoplastic properties of this aggressive subtype of BC tumors. We applied a combination of genetic and pharmacologic manipulations, as well as different biochemical and cell imaging analyses *in vitro*, to investigate the role WAVE3 in the modulation of the CSC phenotype in BC cells. This study also appears to be the first to use CRISPR/Cas9 gene editing to delete WAVE3 in human BC cell lines. Loss WAVE3 in MDA-MB-231 cells with CRISPR/Cas9 resulted in inhibition of cell migration, invasion, invadopodia formation and ECM degradation to levels that surpass those obtained by siRNA- or shRNA-mediated knockdown in our previously published studies [23, 27]. In the present study, we showed that WAVE3 is enriched in the CSC subpopulation of TNBC cells, and depletion of WAVE3 expression in TNBC negatively impacts the CD44^+^/CD24^-^ stem cell population. These reductions are concomitant with inhibition of (i) colony formation of irradiated TNBC cells; (ii) expression of CSCs markers; and (iii) mammosphere formation in serial passages as confirmed by the limiting dilution analysis assay. The regulation of the CSC population by WAVE3 is mediated through its interaction with YB1, a transcription factor that regulates CSC maintenance. Loss of WAVE3 inhibits the nuclear translocation of YB1 and thereby expression of CSCs markers. YB1 is overexpressed in aggressive BC cell lines, both human and mouse, compared to non-tumorigenic or less aggressive cell lines; and the levels of YB1 correlates with the metastatic potential of these cells, as well as correlates with poor prognosis in patients with BC. These associations of YB1 mirror those attributed to WAVE3 [14, 35].

YB1 is a member of the cold-shock protein superfamily and contains a highly conserved nucleic acid-binding motif for both RNA and DNA. In the cytoplasm, YB1 regulates the splicing of pre-mRNA while in the nucleus, it regulates DNA repair, replication and gene transcription [50–53]. Our data show that WAVE3 seems to regulate the nuclear function of YB1. However, WAVE3 can also be found in abundant amounts in the cytoplasm and in the cell membrane, where it regulates actin polymerization and membrane ruffling. Whether WAVE3 is involved in the regulation of YB1 functions in the cytoplasm as well remain to be investigated. There are several parallels with respect to the involvement of WAVE3 and YB1 in promoting the cancer cell phenotype. Both WAVE3 and YB1 are upregulated in BC tumors, and both correlate with poor patient outcome [14, 35, 45]. Mechanistically, both regulate several hallmarks of cancer, including cancer cell proliferation, migration, invasion, metastasis, epithelial-to-mesenchymal transition, tumor angiogenesis, resistance to apoptosis, and chemoresistance [14, 17-23, 27, 35, 37, 47-49, 58-60]. The present study shows for the first time how these two cancer promoter genes collaborate to regulates yet another hallmark of cancer; e.g. the cancer stem cell phenotype. Traditionally, members the WASP/WAVE family have been associated with the remodeling of the actin cytoskeleton thereby regulating the dynamics of lamellipodia and fillopodia and cell membrane ruffles [17, 26, 41, 57, 63]. Our data also identifies a novel role for WAVE3 in the nucleus, where it facilitates the nuclear translocation of YB1 and its transcriptional regulation of CSC-specific genes coupled to their self-renewal and expansion in breast cancer. Thus, we established a WAVE3:YB1 interaction as a key regulator of the CSC phenotype in BC. Therefore we postulate that developing and implementing WAVE3-targeted therapeutics may provide an innovative two-pronged approach to alleviate TNBCs by (i) inhibiting their acquisition of EMT and metastatic phenotypes, and (ii) sensitizing CSCs to therapeutic regimens.

## MATERIALS AND METHODS

### Cell Lines and reagents

Human MCF7, MCF10A, MDA-MB-231, T47D, SKBr3 and BT549 cells, and normal murine mammary gland cells (NMuMG), murine 4T1 cells were obtained from American Type Culture Collection (ATCC). Human MCF10Ca1h and MCF10Ca1a, and murine 67NR and 4T07 were obtained from Dr. Fred Miller (Wayne State University). Cells were maintained in Dulbecco's modified Eagle's medium supplemented with 10% FBS. We used STR DNA fingerprinting analysis for regular authentication of BT549 and MDA-MB-231 cells that we routinely used for this study. Other cell lines were authenticated upon purchase from the ATCC. WAVE3-deficient and YB1-deficient cells were produced by pLenti-CRISPRv2 lentiviral transduction using a scrambled sgRNA (i.e., nonsilencing sgRNA) or two independent and verified WAVE3-specific and YB1-specific sgRNAs for human WAVE3 and YB1 (See Supplementary Table 1 for sgRNA sequences). Stable knockdown of WAVE3 in BT549 was achieved through transfection of BT549 BC cells with either the control non-targeting shRNA or the WAVE3 MISSION shRNA clones (Sigma; Supplementary Table 1) followed by puromycin selection of positive clones as previously described [23, 27]. Stable pools of WAVE3- or YB1-deficient cancer cells were obtained by culture over 14 days in puromycin (5μg/ml). The extent of WAVE3 and YB1 deficiency was determined by immunoblots.

### Antibodies and reagents

The following primary antibodies were from Cell Signaling: Anti-WAVE3, anti-YB1, anti-Lamin-B1 and anti-α-Tubulin. Anti-β-Actin was from Sigma, while living colors anti-GFP and anti-mouse GFP were from Clontech and Santa Cruz Biotechnology Inc., respectively. All primary antibodies were used in 1:1,000 dilution. Goat horseradish peroxidase-conjugated anti-mouse IgG (1:2,000) and goat horseradish peroxidase-conjugated anti-rabbit IgG (1:2,000) were from Calbiochem. Vecta-shield with 4′,6-diamidino-2-phenylindole was from Vector Laboratories. All gel electrophoresis reagents were from BioRad.

### CRISPR/Cas9 gene editing-mediated targeting of WAVE3 and YB1 in cancer cells

LentiCRISPRv2 lentiviral plasmid system (Addgene) was used to specifically knockout WAVE3 and YB1 in human MDA-MB-231 BC cells as described by Cong et al. [33]. The WAVE3 and YB1 specific single guide RNAs (sgRNAs) were identified based on two different predictive algorithms (Chopchop: https://chopchop.rc.fas.harvard.edu; and CRISPR Design, http://crispr.mit.edu). The sgRNAs common to both algorithms were validated against human and mouse GECKOv2 sgRNA libraries [33] and only sgRNAs found in the GECKOv2 libraries were selected. sgRNA oligos were purchased from IDT-DNA and subcloned in lentiCRISPRv2 plasmid [33]. Lentivirus production and cancer cell infection were performed as previously described [64].

### Immunoblot analysis

Whole cell lysates containing similar amounts of total protein (∼25 μg) were resolved on a 4-24% gradient sodium dodecyl sulfate-polyacrylamide gel (BioRad), followed by transfer to Immobilon-P (Millipore) membranes using the Bio-Rad gel and transfer apparatus. Membranes were incubated in 5% whole milk or bovine serum albumin for 1 hour at room temperature, washed with phosphate-buffered saline (PBS), followed by incubation with the primary antibody (as specified) overnight at 4°C. Membranes were then washed and incubated in the appropriate secondary antibody at room temperature for 1 hour, and immunocomplexes were visualized using the Pierce ECL Western blot chemiluminescence detection kit from Thermo Scientific. Signals were quantified using the ImageJ software according to the parameters described in ImageJ user guide (http://rsbweb.nih.gov/ij/docs/guide/146.html). Average values from 3 different blots are presented.

### Immunofluorescence and confocal microscopy

Cells were grown on glass coverslips and fixed in 4% paraformaldehyde for 20 min in PBS at room temperature and washed with PBS. The cells were then permeabilized in 0.2% Triton X-100 in PBS for 15 min, washed again with PBS, and incubated in the blocking solution containing 5% bovine serum albumin (Sigma) in PBS for 2 h at room temperature. Primary as well as secondary antibodies were diluted to the recommended concentration in 5% bovine serum albumin in PBS. Cells were incubated with the primary antibody for 1 h, washed with PBS, and then incubated with the secondary antibody for 1 h. Actin filaments (F-actin) were stained with rhodamine-conjugated phalloidin (Molecular Probes, Eugene, OR) in PBS. The coverslips were mounted on object slides using Vectashield mounting medium containing 4′,6-diamidino-2-phenylindole (Vector Laboratories, Burlingame, CA). Fluorescence images were captured using a Nikon TE2000-E inverted microscope. Signals were quantified using the ImageJ software according to the parameters described in ImageJ user guide (http://rsbweb.nih.gov/ij/docs/guide/146.html). Average values of 5 different images were plotted.

### Invadopodia formation and ECM degradation assays

FITC-gelatin degradation assays were performed as per the manufacturer’s procedure (Invitrogen). In brief, coverslips (18-mm diameter) were coated with 50 ug/ml poly-L-lysine for 20 min at room temperature, washed with PBS, fixed with 0.5% glutaraldehyde for 15 min and washed with PBS for 3 times. After washing, the coverslips were inverted on a drop of 0.2% FITC conjugated gelatin in PBS containing 2% sucrose, incubated for 10 min at room temperature, washed with PBS for 3 times, quenched with sodium borohydride (5 mg/ml) for 3 min and finally incubated in 2 ml of complete medium for 2 h. Cells (2 × 10^5^ each well) were plated in FITC gelatin-coated coverslips and incubated at 37°C for 12 h. Invadopodia and ECM degradation were documented using fluorescence confocal microscopy as described [65]. Signal analysis and reconstruction of 3D images were performed using the ImageJ software.

### Tumorsphere (mammosphere) and extreme limiting dilution assays

Tumorsphere assays were executed as described previously [66] with slight modification. Briefly, single cell suspensions of parental (*i.e.,* Scram) and WAVE3-deficient MDA-MB-231 or BT549 cells were prepared and plated in 5 individual wells/cell line (100 cells/well) in a 96-well, ultra-low attachment plates (Corning). The cultures were fed every 3-4 days with serum-free DMEM (Invitrogen) supplemented with bFGF (20 ng/ml; Invitrogen), EGF (20 ng/ml; Invitrogen), B27 (Life Technologies), and heparin (4 mg/ml; Sigma), and the resulting tumorspheres were enumerated on day 14 by light microscopy. Afterward, the primary tumorspheres for each experimental condition were combined and collected by gentle centrifugation, and subsequently were disrupted by trypsinization and serially passaged across 5 wells/cell line for an additional 14 days to assess the formation of secondary tumorspheres. The process was repeated in its entirety to assess the formation of tertiary tumorspheres.

The impact of WAVE3-deficiency on CSC frequency was also quantified using the extreme limiting-dilution assays (ELDA) as follows. Parental and WAVE3-deficient MDA-MB-231 or BT549 cells were sorted by flow-cytometry into 8 wells of a 96-well, ultra-low attachment plate at the following densities: 1, 5, 10, 20, 40, and 60 cells/well (100 ml). As above, the tumorspheres were fed every 3-4 days and quantified on day 14 by light microscopy. CSC frequencies were calculated using the ELDA Analysis Software as described (http://bioinf.wehi.edu.au/software/elda/index.html; [67]).

### Flow cytometry and cell sorting

Parental and WAVE3-deficient MDA-MB-231 and BT549 cells were harvested using Accutase Cell Detachment Solution according to the manufacturer’s instructions (BD Biosciences), and subsequently were washed in PBS, pelleted, and resuspended in FACS Buffer at a concentration of 1x10^6^ cells/ml. The cells (300 μl/tube) were protected from light and stained on ice for 60 min with PE-conjugated anti-CD24 (1:100 dilution) and APC-conjugated anti-CD44 (1:100 dilution) antibodies. Afterward, the cells were washed twice in FACS buffer prior to analyzing cell surface expression of CD24 and CD44 on a BD Biosciences Aria scanner. In some experiments, “stem” (*i.e.,* CD44^High^/CD24^Low^) and “non-stem” (*i.e.,* CD44^Low^/CD24^High^) cell populations were sorted and collected by FACS, and subsequently were harvested to isolate total RNA to monitor differences in WAVE3 expression by real-time PCR as described below.

### Plasmid construction and transfections

WAVE3 constructs were generated using the PCR from the template WAVE3 cDNA IMAGE clone 4838122 (ATTC) as described previously [19]. Briefly, the ATG sequence for the first methionine was replaced by a TTG. Amplified fragments were subcloned into the pCR2·1 vector using the T-A cloning kit (Invitrogen). The subcloned fragments were inserted into EcoRI-digested pEGFPC2 vector (Clontech), in-frame with the 3′ terminus of EGFP (GFP). All constructs were sequence-verified using a 3100 Genetic Analyzer (ABI Prism). The EGFP-recombinant vectors were used for either transient or stable transfections using standard protocols, and the correct size of the fusion proteins were verified by western blot analysis. Oligonucleotide primers used for PCR and cloning were from SABiosciences and are available upon request.

### Clonogenic cell survival assay

Equal number of cells (1,000 cells) were plated overnight in 12-well plates in triplicates in complete culture medium. Next day, cells were irradiated by using a ^137^cesium cell irradiator at doses of 2, 4, or 6 Gy. Control cells were sham-irradiated. Cells were cultured for a period of 14 days to allow individual colonies to grow. Fresh culture medium was supplemented every 3 days. The resulting colonies were stained with crystal violet and counted under the microscope, and the data were plotted as the average of surviving colonies under each irradiation dose.

### Co-immunoprecipitation and mass-spectroscopy analysis

GFP Co-IP assays were performed using GFP–nano-Antibodies (Allele Biotechnology) according to the manufacturer instruction. After 2h at 4°C, the precipitates from Co-IP were collected by centrifugation, washed and boiled in Laemmli sample buffer. The eluates were then analyzed on 4-20% gradient acrylamide gels under reducing conditions. For the protein digestion, the bands were cut from the gel and washed/destained in 50% ethanol, 5% acetic acid. The gel pieces were then dehydrated in acetonitrile and dried. In-gel tryptic digestion was carried out overnight at room temperature using a 10 ng/μl solution of trypsin (Promega, Madison, WI, USA) in 50 mM NH_4_HCO_3_. Tryptic peptides resulting from the digestion were extracted from the polyacrylamide, combined, evaporated and resuspended in 1% acetic acid. The peptides were subjected to liquid chromatography-electrospray ionization-tandem mass spectrometry (LC-MS/MS) using Finnigan LTQ linear ion trap mass spectrometer system. Ten μl volumes of the extract were injected and the peptides eluted from the Phenomenex Jupiter C18 reversed-phase capillary chromatography column by an acetonitrile/0.1% formic acid gradient at a flow rate of 0.25 μl/min were introduced into the source of the mass spectrometer on-line. The digest was analyzed using the data dependent multitask capability of the instrument acquiring full scan mass spectra to determine peptide molecular weights and product ion spectra to determine amino acid sequence in successive instrument scans. The data were analyzed by using all CID spectra collected in the experiment to search the NCBI non-redundant database with the search program Mascot using a human taxonomy filter. All matching spectra were verified by manual interpretation.

### Real-time quantitative RT-PCR

Total RNA was extracted from cancer cell lines or tumor tissue using TRIzol reagent (Invitrogen), following the manufacturer's instructions. cDNA was generated and used as a template for qRT-PCR as described previously [68]. Oligonucleotide primers used for qRT-PCR were from SABiosciences (Supplementary Table 1).

### Cell migration, invasion and proliferation

Cell migration was assessed by wounding confluent cultures with a micropipette tip and immediately placing them in complete medium. Bright-field images were obtained immediately after wounding and after 18h. Wound closure was quantified by measuring wound areas from ≥6 different fields using ImageJ 1.34s (NIH). For invasion assays, modified Boyden chambers were coated with Matrigel (1:10 dilution; BD Biosciences), the invasion of MDA-MB-231 cells and their WAVE3-deficient derivatives in response to 10% serum was measured as described [35]. Alterations in cell proliferation of MDA-MB-231 cells and their WAVE3-deficient derivatives were determined by counting the number of viable cells. Cells were seeded into six well-plates in complete growth medium, then harvested at one day intervals over 5 days, and counted in a hemocytometer. Cell viability was assessed using trypan blue staining. Assays were performed in triplicates, and the values plotted were the average of two independent experiments.

### Kaplan-meier overall survival analysis

The effect of YB1 expression on the prognosis of 3554 breast cancer patients was analyzed using the Kaplan-Meier plotter online software (http://kmplot.com/analysis/). The Kaplan-Meier plotter evaluates the effect of 54,675 genes on survival using 10,188 cancer samples including breast, lung, ovarian and gastric cancer patients. Kindlin-2 expression and survival data was derived from Affymetrix microarray data (ID: 208628_s_at). To analyze the prognostic value of YB1 gene, the samples were divided into two groups according to the median expression of YB1. The two patient groups (high and low expression of YB1) were compared using the Kaplan-Meier survival plot. The hazard ratio (HR) with 95% confidence intervals (CI), and the log rank *p* value was computed as part of the Kaplan-Meier plotter online software.

### Statistical analyses

The data are presented as means ± standard deviations of at least three independent experiments. The results were tested for significance using an unpaired Student’s *t* test. A *p* value less than 0.05 was considered significant.

## SUPPLEMENTARY MATERIALS FIGURES AND TABLES



## Abbreviations

CSCs: Cancer stem cells
TNBC: Triple negative breast cancer
YB1: Y-box binding protein 1
ELDA: Extreme limited dilution assay
