# Supplementary material for: The WAVE3-YB1 interaction regulates cancer stem cells activity in breast cancer
Source: Oncotarget. 2017 Oct 24;8(61):104072–89. doi: 10.18632/oncotarget.22009 (PMC5732788; doi:10.18632/oncotarget.22009)
Supplement: Supplementary file 1 [file oncotarget-08-104072-s001.pdf]

The WAVE3-YB1 interaction regulates cancer stem cells activity in breast cancer

SUPPLEMENTARY MATERIALS

Nucleotide sequence

```
         WAVE3-WT  TAAGCCACCGCCTCTGAACATCCTGACACCATACAGGTATAGCTTCATGAGTCCCAGAGCC
W3-CRISPR Clone 1  TAAGCCACCGCCTCTGAACATCCTGACAC-ATACAGGTATAGCTTCATGAGTCCCAGAGCC

         WAVE3-WT  TAAGCCACCGCCTCTGAACATCCTGACACCATACAGGTATAGCTTCATGAGTCCCAGAGCC
W3-CRISPR Clone 2  TAAGCCACCGCCTCTGAACATCCTGACACCA---------------------CCCAGAGCC

         WAVE3-WT  TAAGCCACCGCCTCTGAACATCCTGACACCATACAGG---------------------------------TATAGCTTCATGAGTCCCAGAGCC
W3-CRISPR Clone 3  TAAGCCACCGCCTCTGAACATCCTGACACCA------ATATATCTTCATGAGTCCCAGAGCCTCCACCAATATAGCTTCATGAGTCCCAGAGCC

         WAVE3-WT  TAAGCCACCGCCTCTGAACATCCTGACACC-ATACAGGTATAGCTTCATGAGTCCCAGAGCC
W3-CRISPR Clone 4  TAAGCCACCGCCTCCGAACATCCTGACACCAATACAGGTATAGCTTCATGAGTCCCAGAGCC

         WAVE3-WT  TAAGCCACCGCCTCTGAACATCCTGACACCATACAGGTATAGCTTCATGAGTCCCAGAGCC
W3-CRISPR Clone 5  TAAGCCACCGCCTCTGAACATCCTG-----------GTATAGCTTCATGAGTCCCAGAGCC
```

Protein sequence

```
         WAVE3-WT  KPPPLNILTPYRDDKKDGLK
W3-CRISPR Clone 1  KPPPLNILTHTEMTRRMG.
W3-CRISPR Clone 2  KPPPLNILTPR.
W3-CRISPR Clone 3  KPPPLNILTPIYLHESQSLHQR.
W3-CRISPR Clone 4  KPPPPNILTPIQR.
W3-CRISPR Clone 5  KPPPLNILR.
```

Supplementary Figure 1: WAVE3 gene inactivation using CRISPR/Cas9 gene editing. (A) Nucleotide sequence alignment of wild-type (WT) exon 2 of human WAVE3 and representative WAVE3-deficient clones showing Indels. Insertions are shown with Red sequences and deletions sequences are dashed). sg-RNA-1 sequence is underlined and the Protospacer Adjacent Motif (PAM) sequence is highlighted. **(B)**. Protein sequence alignment of WT exon-2 of the human WAVE3 and representative WAVE3-deficient clones showing premature stop codons.

Mascot Search Results

Protein View: **SB12-86-26; Ln8 area 8 ID 10**

Match to: **gi|34098946** Score: **135**
nuclease sensitive element binding protein 1 [Homo sapiens]
Found in search of C:\Xcalibur\data\11sep2613.RAW

Nominal mass (M_r): **35903**; Calculated pI value: **9.87**
NCBI BLAST search of gi|34098946 against nr
Unformatted sequence string for pasting into other applications

Fixed modifications: Carbamidomethyl (C) Variable modifications: Oxidation (M)
Cleavage by Trypsin: cuts C-term side of KR unless next residue is P Sequence Coverage: **5%**

Matched peptides shown in **Bold Red**

1 MSSEAETQQP PAAPPAAPAL SAADTKPGTT GSGAGSGGPG GLTSAAPAGG
51 DKKVIATKVL GTVKWFNVRN GYGFINRNDT KEDVFVHQTA IKKNNPRKYL
101 RSVGDGETVE FDVVEGEK**GA EAANVTGPGG VPVQGSK**YAA DRNHYRRYPR
151 RRGPPRNYQQ NYQNSESGEK NEGSESAPEG QAQQRRPYRR RRFPPYYMRR
201 PYGRRPQYSN PPVQGEVMEG ADNQGAGEQG RPVRQNMYRG YRPRFRRGPP
251 RQRQPREDGN EEDKENQGDE TQGQQPPQRR YRRNFNYRRR RPENPKPQDG
301 KETKAADPPA ENSSAPEAEQ GGAE

Residue Number Increasing Mass Decreasing Mass

Start - End	Observed	Mr(expt)	Mr(calc)	Delta	Miss	Sequence	
119 - 137	848.62	1695.23	1694.86	0.37	0	K.GAEAANVTGPGGVPVQGSK.Y	(Ions score 82)
119 - 137	848.65	1695.28	1694.86	0.42	0	K.GAEAANVTGPGGVPVQGSK.Y	(Ions score 93)

Supplementary Figure 2: Identification of YB1 as a WAVE3-interacting protein using Mass Spectrometry analysis. Mascot search results Mascot match results and the sequences of matched peptides of YB1 protein that were identified via MS/MS. The NCBI non-redundant database was searched using Mascot with a human taxonomy filter.

cNLS Mapper Result
WAVE3

Predicted NLSs in query sequence

```
MPLVKRNIEPRHLCRGALPEGITSELECVTNSTLAAIIRQLSSLSKHAED  50
IFGELFNEANNFYIRANSLQDRIDRLAVKVTQLDSTVEEVSLQDINMKKA 100
FKSSTVQDQQVVSKNSIPNPVADIYNQSDKPPPLNILTPYRDDKKDGLKF 150
YTDPSYFFDLWKEKMLQDTEDKRKEKRRQKEQKRIDGTTREVKKVRKARN 200
RRQEWNMMAYDKELRPDNRLSQSVYHGASSEGSLSPDTRSHASDVTDYSY 250
PATPNHSLHPQPVTPSYAAGDVPPHGPASQAAEHEYRPPSASARHMALNR 300
PQQPPPPPPPQAPEGSQASAPMAPADYGMLPAQIIEYYNPSGPPPPPPPP 350
VIPSAQTAFVSPLQMPMQPPFPASASSTHAAPPHPPSTGLLVTAPPPPGP 400
PPPPPGPPGPGSSLSSSPMHGPPVAEAKRQEPAQPPISDARSDLLAAIRM 450
GIQLKKVQEQREQEAKREPVGNDVATILSRRIAVEYSDSDDDSEFDENDW 500
SD                                                 502
```

Supplementary Figure 3: cNLS Mapper-mediated prediction of two nuclear localization signal sequence in WAVE3 (Red sequences).

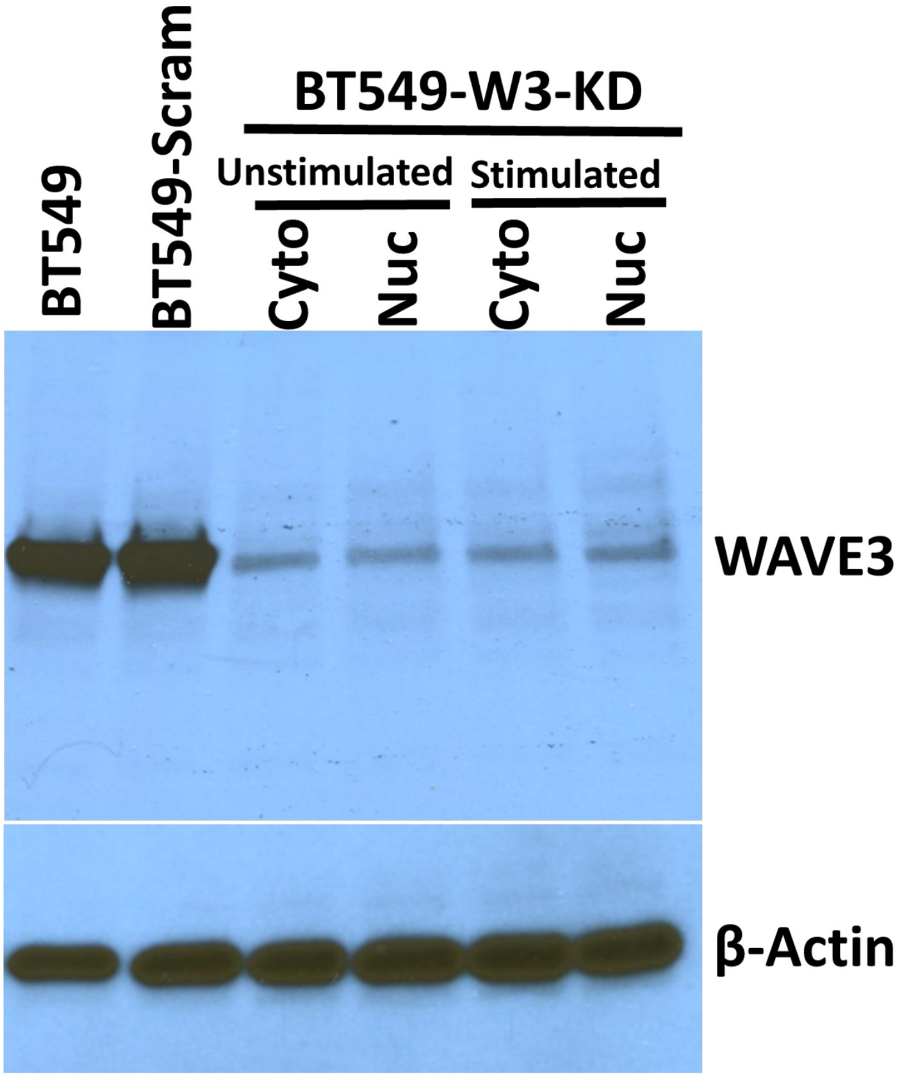

Supplementary Figure 4: In support of Figure 4E. Western blots developed with anti-WAVE3 antibody of protein lysates from parental or control (Scram) BT549 cells, and unstimulated or FBS-stimulated cytosolic (Cyto) or nuclear (Nuc) fractions of WAVE3-deficient (W3-Sh-1) BT549 cells. β-Actin is a loading control.

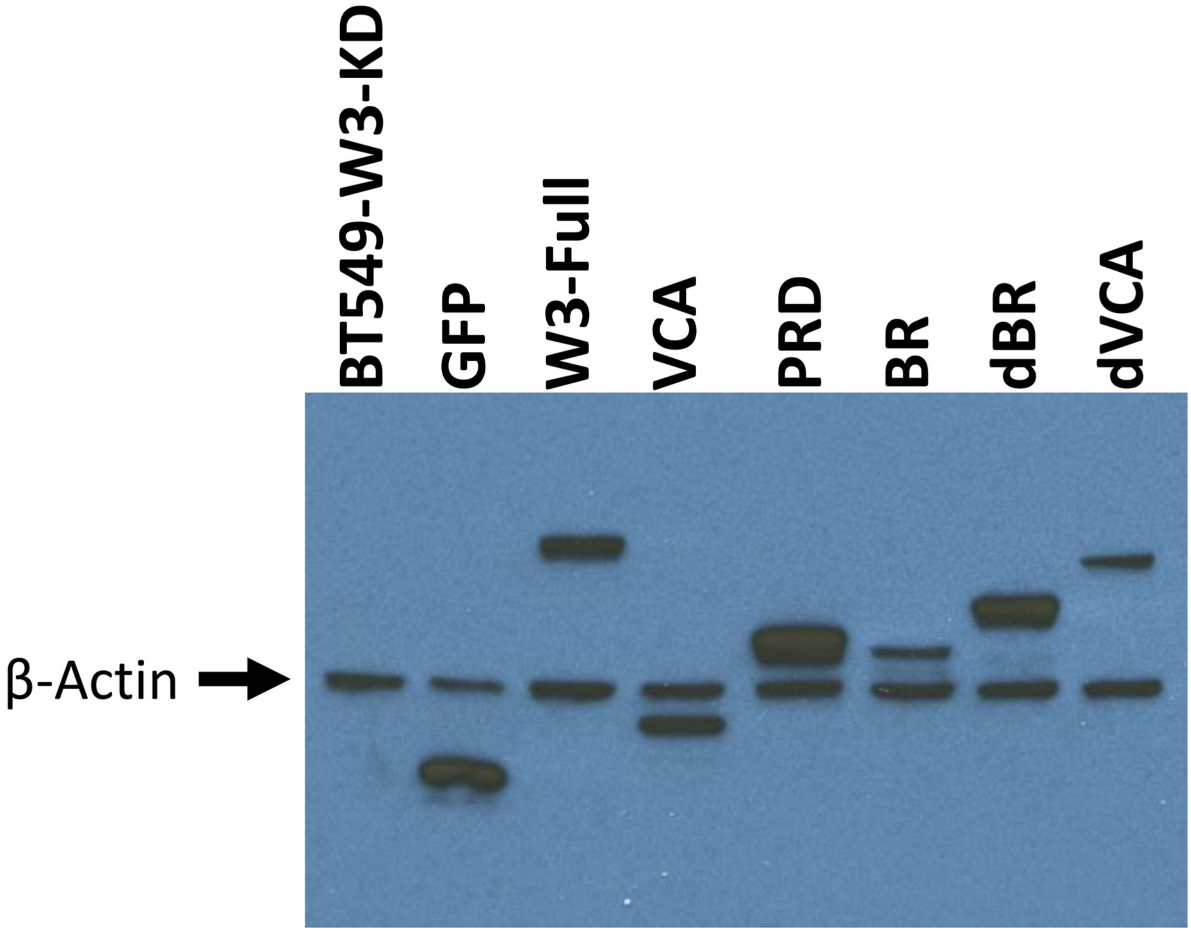

Supplementary Figure 5: In support of Figure 7. Western blots developed with anti-GFP antibody of protein lysates WAVE3-knockdown BT549 cells that were transfected with the indicated expression constructs.

Supplementary Table 1: Primer sequences used in PCR analysis

WAVE3-F: 5'-TGACACCATACAGAGATGAC-3'
WAVE3-R: 5'-CTGTTAGGGTTCAGCTTGTG-3'
YB1-F: 5'-TAGACGCTATCCACGTCGTAG-3'
YB1-R- 5'CCCCACTCTCACTATTCTGGT-3'
NANOG-F: 5'-CCCAAAGGCAAACAACCCACTTCT-3'
NANOG-R: 5'-AGCTGGGTGGAAGAGAACACAGTT-3'
SOX-2-F: 5'-CACATGAAGGAGCACCCGGATTAT-3'
SOX-2-R: 5'-GTTCATGTGCGCGTAACTGTCCAT-3'
OCT4-F: 5'-ATGCATTCAAACTGAGGTGCCTGC-3'
OCT4-R: 5'-AACTTCACCTTCCCTCCAACCAGT-3'
GAPDH-F: 5'-CTGAGTACGTCGTGGAGTC-3'
GAPDH-R: 5'-CAGAGATGATGACCCTTTTG-3'
**Oligonucleotide Sequence of sgRNAs used for CRISPR/Cas9 gene knockdown**
WAVE3-sgRNA-1: 5'-TGCTGTCAAAGTCACCCAGC-3'
WAVE3-sgRNA-2: 5'-TGAACATCCTGACACCATAC-3'
YB1-sgRNA-1: 5'-GGACAAGAAGGTCATCGGTG-3'
YB1-sgRNA-2: 5' GTCTTGCAGGAATGACACCA-3'

WAVE3 MISSION shRNA clones from Sigma

Clone Name	Clone Symbol	Target Sequence
NM_006646.4-3644s1c1	WAVE3-Sh-1	GCCTACTACATTGGCGCTATT
NM_006646.4-294s1c1	WAVE3-Sh-2	CCAGCGAACTTGAATGTGTAA

Supplementary Table 2: List of WAVE3-binding proteins that were identified by immunoprecipitation and Mass-Spectrometry

Protein name	Accession #
GFP	262348071
WAVE3	13699803
WAVE Complex	
WAVE3	13699803
Abl-interactor 1 isoform a	61743942
Cytoplasmic FMR1 interacting protein	2 82617630
NCK-associated protein 1 isoform 1	7305303
Actin	4501885
Actin-related protein 2 isoform b	5031571
Transcription machinery	
Y-Box-binding Protein 1 (YB1)	154355000
General Transcription Factor Ii-I Isoform 1	14670350
Elongation Factor 1-	4503481
Elongation factor 2	4503483
Exportin 1	4507943
Importin 5	24797086
Nucleophosmin 1	10835063
Centrosome Stabilization	
DNA replication licensing factor MCM6	7427519
Others	
ATP synthase subunit alpha, mitochondrial precursor	4757810
nuclease sensitive element binding protein 1	34098946
S-adenosylmethionine synthase isoform type-2	5174529 44
fructose-bisphosphate aldolase A	4557305
fructose-bisphosphate aldolase C	4885063 40
guanine nucleotide-binding protein subunit beta-2-like 1	5174447
ATP-dependent RNA helicase A	100913206
heat shock protein 90kDa beta, member 1	4507677 93
heat shock 70 kDa protein 1A/1B	167466173
